# Association between high-density-lipoprotein cholesterol and postoperative recovery from lumbar disc herniation

**DOI:** 10.1371/journal.pone.0351788

**Published:** 2026-07-24

**Authors:** Nuoman Han, Wei Chen, Shuai Zhou, Rui Chen, Guangzhao Hou, Qian Xiao, Zhenbang Yang, Shihang Liu, Yingze Zhang, Hongzhi Lv

**Affiliations:** 1 Hebei Orthopaedic Research Institute, Department of Orthopaedic Surgery, Hebei Medical University Third Hospital, Shijiazhuang, P.R. China; 2 School of Public Health, Hebei Medical University, Shijiazhuang, P.R. China; 3 Engineering Research Center of Orthopedic MinimallyInvasive Intelligent Equipment, Ministry of Education., Shijiazhuang, P.R China; 4 Key Laboratory of Biomechanics of Hebei Province, Shijiazhuang, P.R. China; 5 NHC Key Laboratory of Intelligent Orthopaedic Equipment, Shijiazhuang, P.R. China; Hokkaido University: Hokkaido Daigaku, JAPAN

## Abstract

**Objective:**

The levels of high density lipoprotein cholesterol (HDL-C) correlate with lumbar spine degeneration, but their value for predicting the prognosis of the lumbar spine is unclear. This study was implemented to explore whether HDL-C levels were associated with postoperative recovery from lumbar disc herniation (LDH).

**Methods:**

Patients with LDH who underwent posterior lumbar interbody fusion (PLIF) admitted between January 1^st^, 2018, and December 31^st^, 2022, were included in this study. The outcome was postoperative recovery as assessed by the economic-functional score. The primary exposure variable was the postoperative plasma HDL-C level. LASSO regression was used to screen for variables associated with outcome, and GAM modeling was used to determine cutoff values for HDL-C levels, with the adjusted model used to estimate the relative contribution of each variable. HDL-C levels were transformed into a dichotomous variable based on the cutoff values, and multifactorial logistic regression was used to assess OR.

**Results:**

A total of 967 patients with LDH were included 529 males (54.7%), 438 females (45.3%), mean age 49.63 ± 12.51 years, mean HDL-C level 1.14 ± 0.25 mmol/L. LASSO regression showed that HDL-C was associated with outcome, there was a risk-protecting effect in the GAM with a cutoff value of 1.15 mmol/L. According to the commonly used clinical classification method, logistic regression showed that normal levels of HDL-C were protective compared with abnormal levels (OR=1.553, 95% CI,1.017–2.372, P = 0.042). HDL-C had a U-shaped effect in patients without cerebral disease, with a range of 1.1–1.8 mmol/L promoting recovery,and in the subgroup without cerebral disease, HDL-C level ranked 6 out of 20 variables, whereas in the overall cohort it ranked 14th and showed no significant effect. Logistic regression showed a protective effect of normal levels of HDL-C over abnormal levels (OR=1.900, 95% CI,1.206–2.992, P = 0.006).

**Conclusion:**

Postoperative HDL-C levels were not significantly associated with 1-year postoperative functional recovery in the overall cohort of patients undergoing PLIF for LDH. In a pre-specified subgroup of patients without cerebral disease, postoperative HDL-C levels exhibited a significant U-shaped association with recovery (optimal range 1.1–1.8 mmol/L). These findings are hypothesis-generating and require validation in future studies.

## Introduction

Lumbar disc herniation (LDH) is a common degenerative lumbar disc disease often associated with low back pain [1]. Low back pain is an extremely common public health problem, of which intervertebral disc degeneration is an important cause [2,3]. The global prevalence of such pain is increasing, and low back pain remains the leading cause of years lived with disability(YLDs) globally, with more than 800 million people worldwide projected to suffer from it by 2050 [4,5]. The social and economic burden of lower back pain is enormous, in terms of both the direct burden of disease and the potential impact [6,7]. Surgery is an important treatment modality for LDH. A retrospective analysis of patients newly diagnosed with back pain or lower limb pain in the United States showed that the annual cost was $265 million for the surgical cohort [8]. How best to evaluate postoperative functional recovery after LDH surgery is also a key issue.

High-density-lipoprotein cholesterol (HDL-C), often referred to as “good cholesterol,” is thought to be associated with a reduced risk of cardiovascular disease [9,10] and to have anti-inflammatory and oxidative stress-inhibiting properties [11]. This makes HDL important in the development and prognosis of many diseases. In recent years, an increasing number of studies have linked lipid levels to the occurrence and prognosis of LDH [12–14]. Several studies have shown that HDL-C can contribute to lumbar arterial atherosclerosis, leading to reduced blood supply to the lumbar spine [15]. HDL-C can also influence prognosis through proinflammatory activators, and the development of endplate inflammation often leads to impaired nutrient exchange and accelerated disc degeneration [16]. A recent genome-wide association study confirmed that HDL-C levels are associated with the risk of reduced lumbar spine bone mineral density (BMD) [17]. These findings are clinically important and suggest that HDL-C levels were associated with the lumbar spine, but there is a need for further evidence of this. Against this background, we hypothesized that HDL-C has an impact on postoperative recovery in patients with LDH. Furthermore, given the established role of apolipoprotein E (ApoE) in both lipid metabolism and central nervous system homeostasis, and its potential influence on intervertebral disc degeneration, we a priori hypothesized that the presence of cerebral disease (defined as cerebrovascular events including cerebral infarction, cerebral hemorrhage, or transient ischemic attack) might modify the association between HDL-C and postoperative recovery. Therefore, we planned a subgroup analysis in patients without cerebral disease to explore this modification effect.

To evaluate the relationship between HDL-C levels and the degree of recovery after LDH, we examined these levels in LDH inpatients after surgical treatment with PLIF to explore whether HDL-C levels impact recovery after LDH and to determine the range of HDL that benefits prognosis. This may provide new insights into the treatment and intervention of complex bone-related features and diseases.

## Methods

### Patient selection

**Fig 1:**
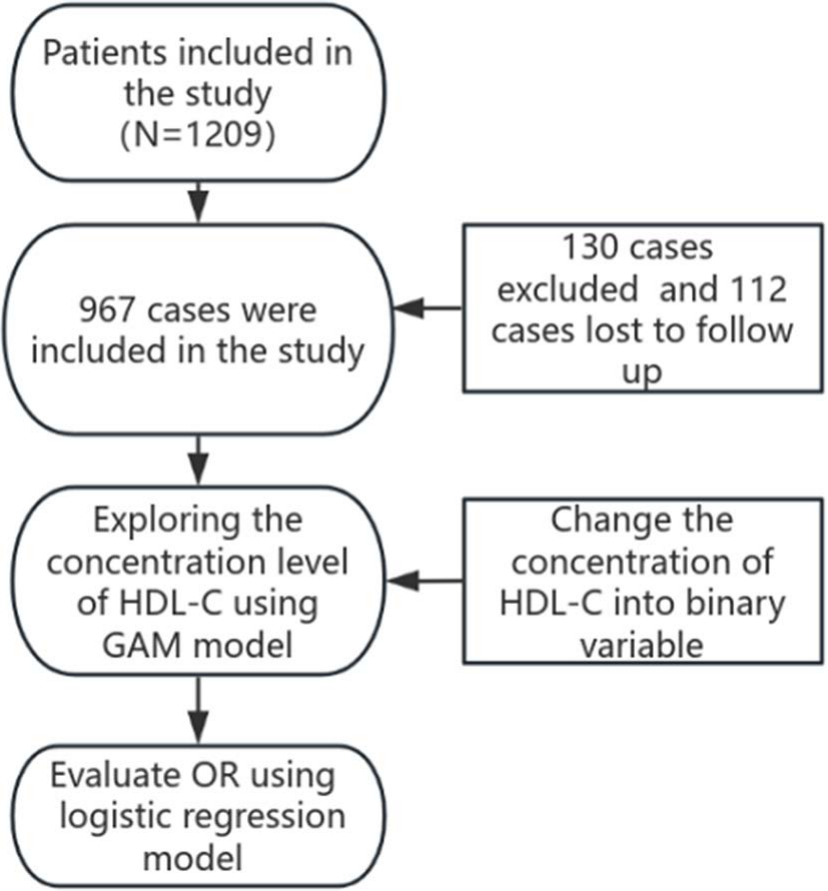
Flow chart of the study.

For this study, clinical data were collected from patients with LDH who were treated with PLIF from January 1, 2018, to December 31, 2022, at the Third Hospital of Hebei Medical University. The data were collected prospectively, using medical records and imaging data. The inclusion criteria were as follows: (1) those who had been clearly diagnosed with disc herniation based on clinical symptoms, signs, and imaging examinations; and (2) those who had been treated with PLIF and recovered for 1 year postoperatively. The exclusion criteria were as follows: (1) patients who were lost to follow-up; (2) those who underwent PLIF and were followed up for at least 12 months after surgery with complete assessment of postoperative recovery outcomes; (3) a history of spinal trauma and intervertebral space infection; (4) incomplete clinical data (basic patient data and surgical data); and (5) under 18 years old (Fig 1). This study was approved by the Ethics Committee of The Third Hospital of Hebei Medical University (K2015-002-1).

### Exposure variable

The primary exposure variable was the HDL-C level (mmol/L). HDL-C was measured in fasting venous blood samples (at least 8 hours of overnight fasting) on the morning of postoperative day 1. This study is a retrospective cohort investigation focusing on the association between postoperative HDL-C level and 1-year postoperative functional recovery, rather than the predictive value of preoperative lipid profiles. Postoperative HDL-C reflects postoperative inflammatory, metabolic, nutritional, and hepatic function, with clear temporal and biological plausibility. This is an observational association study and does not draw causal inferences.

### Data collection

One year after their surgery,data on the following variables were collected through telephone follow-up or record: (1) preoperative factors: sex, age, ethnicity, occupation, marriage, education, body mass index, blood type, season, smoking, alcohol consumption, and common diseases (diabetes mellitus, hypertension, cardiac disease, cerebral disease was defined based on documented clinical diagnosis and/or imaging evidence of cerebrovascular events, including cerebral infarction, cerebral hemorrhage, and transient ischemic attack (TIA), according to the International Classification of Diseases, Tenth Revision (ICD-10) codes I63, I61, and G45, respectively. The term “cerebral disease” is used consistently throughout this study to refer exclusively to these conditions. No other neurological or psychiatric disorders were included in this definition), anemia, liver disease, hypoproteinemia, hypokalemia, osteoporosis); and (2) postoperative factors: hospitalization days, postoperative infection, rehabilitation training, weekly exercise time, and postoperative laboratory indicators: total cholesterol(TC), triglyceride(TG), low density lipoprotein cholesterol(LDL-C). We have dedicated personnel responsible for follow-up, data entry, and other related tasks to ensure quality and consistency in intervention delivery. Since the clinical data involved in this article are secondary uses, the local ethics committee exempted the requirement of informed consent for the use of patient data.

Dates when data were accessed for research purposes 01/01/2024 to 31/03/2024.

### Outcomes

Our primary outcome was postoperative recovery. The A rating scale proposed by Prolo et al., the anatomic–economic-functional rating system (AEF), was applied to measure recovery [18]. In this study, patient condition was assessed 1 year after surgery, and the anatomical evaluation of the scale was not assigned a specific score; therefore, recovery was evaluated according to the economic–functional scoring system: excellent, 9–10 points; good, 7–8 points; moderate, 5–6 points; and poor, 2–4 points. Excellent and good grades were defined as favorable postoperative recovery, while moderate and poor grades were defined as unfavorable recovery. No patients were classified into the moderate or poor groups in the present study. The Prolo economic-functional scale was selected because it is specifically designed for postoperative evaluation of lumbar spine surgery, with good validity and reliability in assessing clinical function and economic recovery. It has been widely adopted in posterior lumbar interbody fusion (PLIF) prognosis studies and enables consistent and semiquantitative evaluation across patients.

### Statistical analysis

All factors initially collected as categorical variables or transformed into such variables were statistically described as frequencies and proportions and compared using the χ^2^ test or Fisher’s exact test. Continuous covariates were expressed as mean ± standard deviation or median depending on whether the data were normally distributed and compared using one-way ANOVA or the Kruskal–Wallis H test, as appropriate. Prior to any regression analysis and modeling, we tested for multicollinearity between all variables by examining the variance inflation factor (VIF), with VIF > 5 being considered to indicate multicollinearity.

Least absolute shrinkage and selection operator (LASSO) regression was used to select candidate variables associated with postoperative functional recovery to avoid overfitting [19]. The penalty parameter (lambda) was determined using 10-fold cross-validation with the minimum mean-squared error as the selection criterion. Variables with non-zero coefficients after penalty tuning were retained for subsequent modeling. Generalized additive models (GAM) were applied to explore the nonlinear association between continuous HDL-C levels and postoperative recovery. The optimal cutoff values of HDL-C were determined based on inflection points in the smoothed curve fitted by the GAM. In the adjusted GAM, continuous variables were fitted using penalized thin-plate regression samples, with categorical variables as factors. Maximum likelihood (ML) methods were used and the model framework was specified as a dichotomous distribution. The relative contribution of each variable was calculated using the variance (χ^2^) minus its df (estimate of the spline term) [20]. Higher values indicate greater contributions.

Multivariate logistic regression models were used to assess the association between HDL-C levels (dichotomized by GAM-determined ranges and ranges determined in clinical practice) and postoperative functional recovery after LDH surgery in the whole cohort and in individual subgroups, adjusting for variables with univariate P < 0.20. In subsequent multivariate analysis, P < 0.05 was considered statistically significant.

The following sensitivity analyses were performed: (1) use of the spinal segment operated on as an adjustment variable, (2) use of the number of spinal segment fusions as an adjustment variable, and (3) removal of those with liver disease due to the synthesis of HDL-C in the liver.

In addition, exploratory analyses were performed using logistic regression, adjusting for the same covariates as in the main model, to explore the application of HDL-C level as a dichotomous variable determined by GAM to other groups.

All statistical analyses were performed using R4.4.1 (R Foundation for Statistical Computing, Vienna,Austria) and SPSS 26.0 (IBM,Armonk,NY, USA).

## Result

### Baseline characteristics

A total of 1209 patients were initially assessed for eligibility. Among them, 130 patients were excluded, and 112 patients were lost to follow-up. Finally, 967 patients were included in the final analysis, 529 (54.7%) were male and 438 (45.3%) were female, with mean age of 49.63 ± 12.51 years. According to the economic-functional scoring system, 824 patients (85.21%) achieved an excellent recovery, 143 patients (14.79%) achieved a good recovery, and no patients were classified as moderate or poor (n = 0 for both grades). The HDL-C level was 1.14 ± 0.25 mmol/L. The baseline characteristics of the patients classified according to HDL-C levels and the baseline characteristics of the classification are shown in S1 Table.

**Fig 2:**
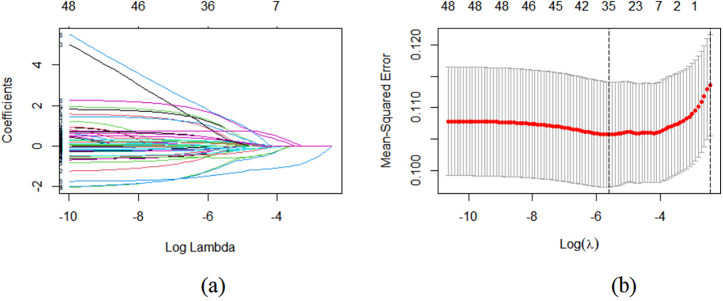
(a) LASSO regression coefficient profiles: displaying the progression of coefficients of various predictors as the regularization parameter (lambda) is increased. Each line represents a different predictor variable in the LASSO regression model. (b) Selection of Lambda in LASSO Regression: This graph shows the cross-validation curve for model tuning. The lambda.min is highlighted, indicating the optimal level of penalization for the LASSO model.

**Fig 3:**
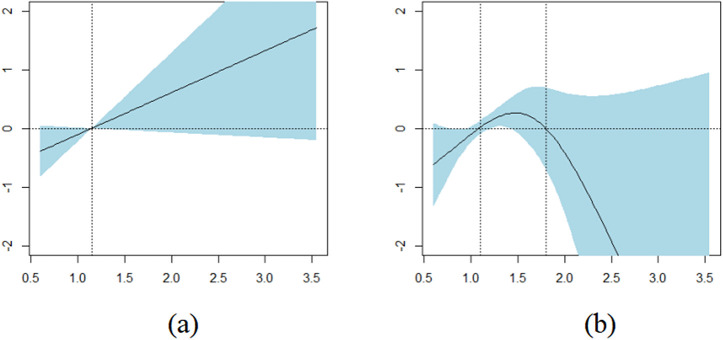
Visualization of the risk for recovery of LDH according to HDL-C based on Generalized Additive Model. (a) Overall group;(b)Non-cerebral diseases group.

There was no multicollinearity between the variables (VIF < 5) (S2 Table). Factors associated with postoperative functional recovery after LDH surgery were screened by LASSO regression (Fig 2) (S3 Table), in which HDL-C level, among others, was shown to have an impact on the results. However, using the GAM, it was found that HDL-C levels did not meaningfully influence the outcome (Fig 3a), at which point the cutoff value was 1.15 mmol/L, and according to the overall adjusted GAM, HDL-C concentration ranked 14^th^ out of the 21 variables (Table 1). Because the results did not significantly affect the outcome, we dichotomized by normal concentration value(HDL-C ≥ 1.1 mmol/L). The dichotomized HDL-C ≥ 1.1 mmol/L in the overall cohort was based on clinical routine cutoff, which was determined before the subgroup nonlinear relationship was observed. In this analysis, HDL-C was found to contribute to postoperative recovery (OR=1.553, 95% CI, 1.017–2.372, P = 0.042) (Table 2). Sensitivity analyses showed that the resultant liver disease would have an impact on the results, and the rest showed that the results remained robust (Table 3).

**Table 1:** Relative contribution of model covariates for recovery based on GAM.

Variable	Overall		Non-Cerebral Diseases Group	
	χ^2^ − *df*	Rank	χ^2^ − *df*	Rank
Sex	0.550	8	−0.079	11
Age	6.377*	4	8.799*	3
Place of Residence	0.251	11	1.800	8
Smoking	20.145*	2	17.695*	2
Alcohol	−0.361	15	−0.375	12
Marriage	−1.000	19	−1.000	16
Season	3.986	5	3.109	5
Blood Type	−0.953	18	−1.462	18
Occupation	0.133	12	−3.384	20
BMI	−2.554	21	−1.765	19
Sport time	25.858*	1	22.602*	1
Rehabilitation	1.024	7	−0.480	13
Hepatopathy	−0.850	16	−0.984	15
Hypokalemia	1.601	6	2.127	7
Anemia	0.113	13	−0.962	14
Infection	−1.000	19	−1.000	16
Osteoporosis	6.929*	3	4.862*	4
Kidney disease	0.382	10	1.250	9
TG	0.513	9	0.995	10
HDL-C	0.046	14	2.769	6
Cerebral disease	−0.876	17	N/A	

†χ^2^ − *df* is the χ^2^ estimate minus the *df* for the variables in the model term.

*Covariate is significant (P < 0.05).

**Table 2:** Adjusted logistic regression analyses on the association of HDL-C and the recovery of LDH in overall group.

Group	Logistic regression model	
	Adjusted OR (95% CI)	P
Model 1	1.553(1.017-2.372)	0.042*

* *P* ＜ 0.05.

Model 1: HDL-C ≥ 1.10 mmol/L was defined as normal level. Adjusted covariates: age, gender, season, length of hospitalization, residence, smoking, alcohol consumption, rehabilitation, weekly exercise time, HDL-C, TG, osteoporosis, heart disease, and hypoproteinaemia.

**Table 3:** Sensitivity analyses for the relationship between HDL-C and the recovery of LDH in overall group.

Group	Logistic regression model	
	Adjusted OR (95% CI)	P
Number of vertebral bodies	1.557 (1.019-2.381)	0.041*
Surgical vertebral bodies	1.547(1.013-2.365)	0.044*
Patients without hepatopathy	1.472(0.945-2.294)	0.087

* *P* ＜ 0.05.

The presence of comorbidities may impact HDL-C levels, so we analyzed comorbidities screened by LASSO regression for those without comorbidities and explored HDL-C concentrations using the GAM. In the pre-specified subgroup of patients without cerebral disease, HDL-C concentration was significantly associated with postoperative recovery (Fig 3b), which was used to determine the cutoff values for its concentration, which were 1.1 and 1.8 mmol/L. According to the overall adjusted GAM, HDL-C concentration ranked 6^th^ out of 20 variables (Table 1). We dichotomized HDL-C levels by determining the cutoff value using the GAM. Multivariate logistic analysis showed that normal levels of HDL-C compared with abnormal levels were protective during postoperative functional recovery after LDH surgery (OR=1.900, 95% CI, 1.206–2.992, P = 0.006) (Table 4). The sensitivity analyses showed that the contribution of HDL-C remained robust (S4 Table).

**Table 4:** Adjusted logistic regression results for the relationship between HDL-C and the recovery of LDH in non-cerebral diseases group.

Group	Logistic regression model			
	Unadjusted OR (95% CI)	P	Adjusted OR (95% CI)	P
Non-Cerebral Diseases	1.804 (1.226-2.653)	0.003*	1.900 (1.206-2.992)	0.006*

* *P* ＜ 0.05

Adjusted covariables in logistic regression: age, sex, season, length of hospitalization, blood type, smoking, alcohol consumption, rehabilitation, weekly exercise time, HDL-C, TG, osteoporosis, heart disease, and hypoproteinemia (P < 0.2).

We applied the cutoff values determined according to Fig 3 to the overall population. The results showed that, when applied to the overall population with the HDL-C concentration determined for patients without cerebral disease (Table 5), it also contributed to recovery (OR=1.639, 95% CI, 1.076–2.495).

**Table 5:** Exploratory analyses on the association of HDL-C and the recovery of LDH in overall group.

Group	Logistic regression model	
	Adjusted OR (95% CI)	P
Model 1	1.473 (0.956-2.272)	0.079
Model 2	1.687 (1.105-2.573)	0.015*

* *P* ＜ 0.05.

Model 1: The normal concentration of HDL-C was defined as ≥1.15 mmol/L；

Model 2: The normal concentration of HDL-C was defined as 1.10≤HDL-C≤1.80mmol/L.

## Discussion

This observational cohort study investigated the association between postoperative HDL-C levels and 1-year functional recovery following PLIF in patients with LDH using GAM. In the overall cohort, HDL-C ranked 14th among variables in the adjusted GAM and was not significantly associated with recovery; however, a U-shaped relationship was observed in the pre-specified subgroup without cerebral disease, with 1.1–1.8 mmol/L as the optimal range for recovery. These findings were robust in sensitivity analyses but should be regarded as hypothesis-generating rather than definitive confirmatory results.

HDL-C exerts anti-inflammatory and anti-oxidative effects and modulates osteoblast and osteoclast function, which may contribute to postoperative tissue repair and bone fusion [11,21]. Most previous studies focused on the association between HDL-C and lumbar bone mineral density [21–25], with limited evidence regarding postsurgical recovery. Our findings extend current knowledge by demonstrating a nonlinear, U-shaped association between postoperative HDL-C and LDH recovery, especially in patients without cerebral disease.

The potential modifying effect of cerebral disease is hypothesized to be related to apolipoprotein E (ApoE), which plays a key role in both lipid metabolism and central nervous system homeostasis [26–29]. ApoE deficiency impairs disc nutrient supply and accelerates degeneration [30,31]. We speculate that cerebral disease may alter ApoE levels, thereby affecting HDL-C function, inflammation, and metabolic balance, ultimately weakening the association between HDL-C and recovery. Notably, ApoE levels were not measured in this study; this mechanistic explanation is purely speculative and requires experimental validation in future research.

Traditionally, higher HDL-C levels were considered cardioprotective [9,10]. However, recent studies suggest a U-shaped or even adverse association with clinical outcomes at very high concentrations, including mortality, fracture risk, and infectious disease [32–36,37]. Our results align with this emerging paradigm: excessive HDL-C did not confer additional benefit, supporting the notion that “higher is not always better.”

In addition to HDL-C, longer weekly exercise time, absence of osteoporosis, and nonsmoking were independently associated with better recovery. These factors promote bone fusion, reduce inflammation, and improve spinal function, consistent with previous literature [38–43].

Several limitations should be acknowledged. First, postoperative outcomes were highly skewed (85.21% excellent, 14.79% good, no moderate/poor cases), which may reduce the discriminatory power of the Prolo economic-functional scale [18]. The excellent outcomes may reflect standardized PLIF, rigorous patient selection, and systematic postoperative rehabilitation in our center. Future studies using more sensitive tools such as ODI or SF-36 are warranted. Second, this was a single-center retrospective study with potential selection bias; large-scale multicenter studies are needed. Third, the cutoff values derived from GAM are hypothesis-generating rather than confirmatory. Finally, residual confounding cannot be excluded.

## Conclusion

In conclusion, this observational retrospective cohort study demonstrated that postoperative HDL-C levels were not independently associated with 1-year functional recovery in the overall cohort after PLIF for LDH. In the exploratory subgroup without cerebral disease, postoperative HDL-C showed a significant U-shaped association with recovery, with an optimal range of 1.1–1.8 mmol/L. This subgroup finding is hypothesis-generating rather than confirmatory and should be interpreted with caution. These findings highlight the importance of postoperative metabolic management and provide a reference for predicting recovery after PLIF.

## Supporting information

S1 Table Baseline characteristics of participants. (DOCX)

S2 Table Multicollinearity Test. (DOCX)

S3 Table Coefficients and lambda.min value of the LASSO regression. (DOCX)

S4 Table Sensitivity analyses for the relationship between HDL-C and the recovery of LDH in non-cerebral diseases group. (DOCX)

## Abbreviations

BMI: Body mass index
OR: Odds ratio
TC: Total cholesterol
TG: Triglyceride
HDL-C: High density lipoprotein cholesterol
LDL-C: Low density lipoprotein cholesterol
VIF: Variance inflation factor
PLIF: Posterior lumbar interbody fusion
