# Supplementary material for: Association between high-density-lipoprotein cholesterol and postoperative recovery from lumbar disc herniation
Source: PLoS One. 2026 Jul 24;21(7):e0351788. doi: 10.1371/journal.pone.0351788 (PMC13399355; doi:10.1371/journal.pone.0351788)
Supplement: S1 Table — (DOCX) [file pone.0351788.s001.docx]

**Supplementary Materials**

Supplementary Table S1. Baseline Characteristics of Participants

Comorbid conditions of patients were determined based on ICD-10 diagnoses.

Quantitative variables:

Normal concentration value of HDL-C:≥1.1mol/L.

Supplementary Table S1. Baseline Characteristics of Participants.

| Variable | All | HDL＜1.1 | HDL≥1.1 | P value |
| --- | --- | --- | --- | --- |
| Economic Function Rationg Score | | | | |
| Excellent | 824 | 367 | 461 | 0.008 |
| Good | 143 | 80 | 63 |  |
| Gender | | | | |
| Male | 529 | 297 | 232 | 0.000 |
| Female | 438 | 146 | 292 |  |
| Age | | | | |
| ＜35 | 132 | 80 | 52 | 0.000 |
| 35-49 | 318 | 155 | 163 |  |
| 50-64 | 391 | 150 | 241 |  |
| >64 | 126 | 58 | 68 |  |
| Occupation | | | | |
| Student | 8 | 5 | 3 | 0.086 |
| Office worker | 51 | 31 | 20 |  |
| Farmer | 285 | 143 | 142 |  |
| Manual worker | 96 | 48 | 48 |  |
| Retired | 49 | 18 | 31 |  |
| Others | 478 | 198 | 280 |  |
| Smoking | | | | |
| Yes | 298 | 185 | 113 | 0.000 |
| No | 669 | 258 | 411 |  |
| Drinking | | | | |
| Yes | 283 | 146 | 137 | 0.020 |
| No | 684 | 267 | 387 |  |
| BMI (kg/m2 ) | | | | |
| <18.5 | 11 | 6 | 5 | 0.192 |
| 18.5–23.9 | 311 | 133 | 178 |  |
| 24–27.9 | 414 | 185 | 229 |  |
| ≥ 28.0 | 231 | 119 | 112 |  |
| Marriage | | | | |
| Single | 38 | 25 | 13 | 0.086 |
| Married | 915 | 412 | 503 |  |
| Widowed | 8 | 3 | 5 |  |
| Divorced | 6 | 3 | 3 |  |
| Blood Type | | | | |
| A | 232 | 105 | 127 | 0.935 |
| B | 353 | 161 | 192 |  |
| O | 297 | 140 | 157 |  |
| AB | 85 | 37 | 48 |  |
| Season | | | | |
| Spring | 240 | 105 | 135 | 0.576 |
| Summer | 298 | 136 | 162 |  |
| Autumn | 269 | 121 | 148 |  |
| Winter | 160 | 81 | 79 |  |
| Residence | | | | |
| Urban | 649 | 294 | 355 | 0.648 |
| Rural | 318 | 149 | 169 |  |
| Hospitalization Days | | | | |
| ≤7 | 166 | 80 | 86 | 0.034 |
| 8-14 | 707 | 309 | 398 |  |
| >14 | 94 | 54 | 40 |  |
| Diabetes Mellitus | | | | |
| Yes | 94 | 46 | 155 | 0.522 |
| No | 873 | 397 | 358 |  |
| Hypertension | | | | |
| Yes | 288 | 132 | 156 | 0.993 |
| No | 679 | 311 | 368 |  |
| Heart Disease | | | | |
| Yes | 178 | 84 | 94 | 0.683 |
| No | 789 | 359 | 430 |  |
| Cerebral Diseases | | | | |
| Yes | 102 | 40 | 62 | 0.111 |
| No | 865 | 403 | 462 |  |
| Hepatobiliary Diseases | | | | |
| Yes | 110 | 52 | 58 | 0.744 |
| No | 857 | 391 | 466 |  |
| Anemia | | | | |
| Yes | 67 | 30 | 37 | 0.860 |
| No | 900 | 413 | 487 |  |
| Hypoproteinemia | | | | |
| Yes | 43 | 27 | 16 | 0.022 |
| No | 924 | 416 | 508 |  |
| Kidney Disease | | | | |
| Yes | 35 | 17 | 18 | 0.739 |
| No | 932 | 426 | 506 |  |
| Hypokalemia | | | | |
| Yes | 79 | 41 | 38 | 0.257 |
| No | 888 | 402 | 486 |  |
| Postoperative Infection | | | | |
| Yes | 10 | 5 | 5 | 0.789 |
| No | 957 | 438 | 519 |  |
| Osteoporosis | | | | |
| Yes | 69 | 21 | 48 | 0.008 |
| No | 898 | 422 | 476 |  |
| Rehabilitation training | | | | |
| Yes | 240 | 93 | 147 | 0.011 |
| No | 727 | 350 | 377 |  |
| Weekly exercise time | | | | |
| 0-7h | 60 | 34 | 26 | 0.000 |
| 8-14h | 246 | 96 | 150 |  |
| 15-21h | 369 | 141 | 228 |  |
| 22-28h | 80 | 43 | 37 |  |
| 29-35h | 104 | 60 | 44 |  |
| ＞35h | 108 | 69 | 39 |  |
| TC | | | | |
| ≤5.2 | 766 | 408 | 358 | 0.000 |
| ＞5.2 | 201 | 35 | 166 |  |
| TG | | | | |
| ≤1.7 | 737 | 314 | 423 | 0.000 |
| ＞1.7 | 230 | 129 | 101 |  |
| LDL-C | | | | |
| ≤3.4 | 846 | 418 | 428 | 0.000 |
| ＞3.4 | 121 | 25 | 96 |  |
| Sum | 967 | 443 | 524 |  |
