# Supplementary material for: Association between high-density-lipoprotein cholesterol and postoperative recovery from lumbar disc herniation
Source: PLoS One. 2026 Jul 24;21(7):e0351788. doi: 10.1371/journal.pone.0351788 (PMC13399355; doi:10.1371/journal.pone.0351788)
Supplement: S2 Table — (DOCX) [file pone.0351788.s002.docx]

**Supplementary Materials**

Supplementary Table S2. Multicollinearity Test

Comorbid conditions of patients were determined based on ICD-10 diagnoses.

Quantitative variables:

Normal concentration value of HDL-C:≥1.1mol/L

Supplementary Table S2. Multicollinearity Test

|  | Unstandardized Coefficients | | Standardized Coefficients | t | Sig. | Collinearity Statistics | |
| --- | --- | --- | --- | --- | --- | --- | --- |
|  | B | Std. Error | Beta |  |  | Tolerance | VIF |
| (Constant) | 1.031 | 0.132 |  | 7.789 | 0.000 |  |  |
| Sex | -0.045 | 0.029 | -0.063 | -1.554 | 0.121 | 0.564 | 1.773 |
| Age | -0.053 | 0.015 | -0.133 | -3.441 | 0.001 | 0.621 | 1.610 |
| Marrige | 0.016 | 0.043 | 0.012 | 0.383 | 0.702 | 0.886 | 1.128 |
| Season | -0.024 | 0.011 | -0.070 | -2.242 | 0.025 | 0.946 | 1.057 |
| Hospitalization | 0.003 | 0.022 | 0.005 | 0.154 | 0.878 | 0.883 | 1.133 |
| Blood type | -0.013 | 0.012 | -0.034 | -1.100 | 0.272 | 0.972 | 1.029 |
| Occupation | 0.007 | 0.007 | 0.029 | 0.914 | 0.361 | 0.935 | 1.069 |
| Residence | 0.028 | 0.023 | 0.037 | 1.197 | 0.231 | 0.972 | 1.029 |
| Smoking | -0.199 | 0.030 | -0.259 | -6.580 | 0.000 | 0.594 | 1.685 |
| Drinking | -0.051 | 0.026 | -0.065 | -1.943 | 0.052 | 0.812 | 1.231 |
| Weekly exercise time | 0.019 | 0.009 | 0.075 | 2.208 | 0.028 | 0.808 | 1.238 |
| Rehabilitation | 0.051 | 0.026 | 0.062 | 1.928 | 0.054 | 0.893 | 1.120 |
| BMI | 0.018 | 0.015 | 0.039 | 1.222 | 0.222 | 0.917 | 1.091 |
| TC | 0.021 | 0.039 | 0.024 | 0.532 | 0.595 | 0.472 | 2.119 |
| TG | 0.049 | 0.027 | 0.059 | 1.822 | 0.069 | 0.870 | 1.150 |
| HDL-C | -0.051 | 0.024 | -0.072 | -2.114 | 0.035 | 0.798 | 1.253 |
| LDL-C | -0.066 | 0.046 | -0.061 | -1.440 | 0.150 | 0.512 | 1.952 |
| Hypertension | -0.009 | 0.026 | -0.011 | -0.336 | 0.737 | 0.812 | 1.231 |
| Diabetes | 0.019 | 0.039 | 0.016 | 0.503 | 0.615 | 0.889 | 1.124 |
| Cerebral disease | 0.005 | 0.041 | 0.004 | 0.114 | 0.909 | 0.923 | 1.083 |
| Heart disease | 0.024 | 0.029 | 0.026 | 0.823 | 0.410 | 0.902 | 1.109 |
| Hepatopathy | 0.003 | 0.035 | 0.003 | 0.095 | 0.924 | 0.954 | 1.049 |
| Hypokalemia | 0.054 | 0.041 | 0.042 | 1.307 | 0.191 | 0.904 | 1.106 |
| Anemia | -0.037 | 0.046 | -0.026 | -0.808 | 0.419 | 0.862 | 1.161 |
| Hypoproteinemia | -0.062 | 0.056 | -0.036 | -1.113 | 0.266 | 0.870 | 1.149 |
| Infection | 0.119 | 0.111 | 0.034 | 1.069 | 0.285 | 0.917 | 1.091 |
| Osteoporosis | -0.121 | 0.044 | -0.088 | -2.770 | 0.006 | 0.916 | 1.092 |
| Kidney disease | 0.087 | 0.059 | 0.046 | 1.472 | 0.141 | 0.954 | 1.048 |

VIF > 5 was considered multicollinearity.
