# Supplementary material for: Association between high-density-lipoprotein cholesterol and postoperative recovery from lumbar disc herniation
Source: PLoS One. 2026 Jul 24;21(7):e0351788. doi: 10.1371/journal.pone.0351788 (PMC13399355; doi:10.1371/journal.pone.0351788)
Supplement: S3 Table — (DOCX) [file pone.0351788.s003.docx]

**Supplementary Materials**

Supplementary Table S3.Coefficients and lambda.min value of the LASSO regression

Comorbid conditions of patients were determined based on ICD-10 diagnoses.

Quantitative variables:

Normal concentration value of HDL-C:≥1.1mol/L.

Supplementary Table S3.Coefficients and lambda.min value of the LASSO regression

| Variable. | Variable.Coefficient | lambda.min  0.003707534 |
| --- | --- | --- |
| Sex | -0.28453911 |  |
| Age | -0.28782653 |  |
| Marriage | -0.09990377 |  |
| Season | -0.10666270 |  |
| Blood Type | 0.10653972 |  |
| Occupation | 0.21589636 |  |
| Residence | 0.12526367 |  |
| Smoking | -1.40236146 |  |
| Drinking | -0.20361057 |  |
| Weekly exercise time | 1.43975257 |  |
| Rehabilitation | -1.01584331 |  |
| BMI | 0.19314830 |  |
| TG | 0.22167955 |  |
| HDL-C | -0.10043503 |  |
| Cerebral disease | -0.10462879 |  |
| Hepatopathy | 0.29511482 |  |
| Hypokalemia | 0.32953782 |  |
| Anemia | -0.39393745 |  |
| Postoperative infection | 1.46973223 |  |
| Osteoporosis | -0.29479849 |  |
| Kidney disease | 0.34645836 |  |
