# Supplementary material for: Association between high-density-lipoprotein cholesterol and postoperative recovery from lumbar disc herniation
Source: PLoS One. 2026 Jul 24;21(7):e0351788. doi: 10.1371/journal.pone.0351788 (PMC13399355; doi:10.1371/journal.pone.0351788)
Supplement: S4 Table — (DOCX) [file pone.0351788.s004.docx]

**Supplementary Materials**

Supplementary Table S4.Sensitivity analyses for the relationship between HDL-C and the recovery of LDH in non-cerebral diseases group

Comorbid conditions of patients were determined based on ICD-10 diagnoses.

Quantitative variables:

Normal concentration value of HDL-C:≥1.1mol/L.

Supplementary Table S4. Sensitivity analyses for the relationship between HDL-C and the recovery of LDH in non-cerebral diseases group

| Group | Logistic regression model | |
| --- | --- | --- |
|  | Adjusted OR (95% CI) | P for interaction |
| Number of infusion vertebral bodies | 1.894 (1.201-2.986) | 0.006* |
| Surgical vertebral  bodies  Patients without hepatopathy | 1.902(1.209 -2.994)  1.574(1.009 -2.456) | 0.005*  0.046* |

* *P*＜0.05
